# Detailed Analysis of Focal Chromosome Arm 1q and 6p Amplifications in Urothelial Carcinoma Reveals Complex Genomic Events on 1q, and *SOX4* as a Possible Auxiliary Target on 6p

**DOI:** 10.1371/journal.pone.0067222

**Published:** 2013-06-18

**Authors:** Pontus Eriksson, Mattias Aine, Gottfrid Sjödahl, Johan Staaf, David Lindgren, Mattias Höglund

**Affiliations:** 1 Department of Oncology, Clinical Sciences, Skåne University Hospital, Lund University, Lund Sweden; 2 CREATE Health Strategic Center for Translational Cancer Research, Lund University, Lund, Sweden; 3 Center for Molecular Pathology, Department of Laboratory Medicine, Skåne University Hospital, Lund University, Malmö, Sweden; Deutsches Krebsforschungszentrum, Germany

## Abstract

**Background:**

Urothelial carcinoma shows frequent amplifications at 6p22 and 1q21–24. The main target gene at 6p22 is believed to be *E2F3*, frequently co-amplified with *CDKAL1* and *SOX4*. There are however reports on 6p22 amplifications that do not include *E2F3*. Previous analyses have identified frequent aberrations occurring at 1q21–24. However, due to complex rearrangements it has been difficult to identify specific 1q21–24 target regions and genes.

**Methods:**

We selected 29 cases with 6p and 37 cases with 1q focal genomic amplifications from 261 cases of urothelial carcinoma analyzed by array-CGH for high resolution zoom-in oligonucleotide array analyses. Genomic analyses were combined with gene expression data and genomic sequence analyses to characterize and fine map 6p22 and 1q21–24 amplifications.

**Results:**

We show that the most frequently amplified gene at 6p22 is *SOX4* and that *SOX4* can be amplified and overexpressed without the *E2F3* or *CDKAL1* genes being included in the amplicon. Hence, our data point to *SOX4* as an auxiliary amplification target at 6p22. We further show that at least three amplified regions are observed at 1q21–24. Copy number data, combined with gene expression data, highlighted *BCL9* and *CHD1L* as possible targets in the most proximal region and *MCL1*, *SETDB1*, and *HIF1B* as putative targets in the middle region, whereas no obvious targets could be determined in the most distal amplicon. We highlight enrichment of G4 quadruplex sequence motifs and a high number of intraregional sequence duplications, both known to contribute to genomic instability, as prominent features of the 1q21–24 region.

**Conclusions:**

Our detailed analyses of the 6p22 amplicon suggest *SOX4* as an auxiliary target gene for amplification. We further demonstrate three separate target regions for amplification at 1q21–24 and identified *BCL9, CHD1L*, and *MCL1*, *SETDB1*, and *HIF1B* as putative target genes within these regions.

## Introduction

Urothelial carcinoma (UC) is the sixth most common malignancy and the fourth most common cancer among males. UC originates from the epithelial cells of the inner lining of the bladder wall. Most tumors (70%) are papillary and confined to the urothelial mucosa (stage Ta) or to the lamina propria (stage T1), whereas the remaining are muscle invasive (T2–T4). Most Ta tumors are of low grade, rarely progress, and are associated with a favorable prognosis whereas high grade Ta (TaG3) and T1 tumors have a significant risk of tumor progression. UC has been studied by gene expression profiling [1]–[6] and recently Lindgren et al. [7] classified UC based on gene expression and genomic alterations. Several genes are known to be mutated in UC, of which activating mutations in *FGFR3* and inactivating mutations in *TP53* are the most frequent. Accumulated data has shown that *FGFR3* mutations are characteristic for low grade and low stage tumors whereas *TP53* mutations are characteristic for invasive tumors [8]–[10]. Apart from gene mutations, cytogenetic studies have revealed several recurring chromosomal changes and comparative genome hybridization (CGH) methods have corroborated many of these findings, but also defined several recurrent high level amplifications and deletions [7], [11]–[19]. Key findings of these investigations are frequent losses of chromosome arms 9p and 9q, and frequent amplifications on 6p and 1q. Losses of chromosome 9, and of 9p in particular, are highly characteristic for low stage and low grade UC. Deletions affecting 9p are commonly attributed to loss of the tumor suppressor gene *CDKN2A* at 9p21 [20]. High-level amplifications on 6p are commonly localized to the 6p22.3 region and are frequent in advanced stage UC. The genes most frequently encompassed by 6p22 amplifications are *E2F3*, *CDKAL1*, and *SOX4*. Amplifications at 1q21–24 are frequent but heterogeneous. The heterogeneity of 1q21–24 amplifications has most likely precluded the identification of bona fide target genes. In order to clarify some of the genomic features of 6p and 1q amplifications in UC we have applied high-resolution array CGH focused at regions commonly altered in UC combined with gene expression analysis.

## Materials and Methods

### Patients and tumor tissue samples

Samples were obtained by cold-cup biopsies from the exophytic part of the bladder tumor from patients undergoing transurethral resection at hospitals of the Southern Healthcare Region of Sweden. Pathological evaluation was based on WHO 1999. Written informed consent was obtained from all patients and the study was approved by the Local Ethical Committee at Lund University. Using previous information on genomic imbalances in 261 cases of urothelial carcinoma [3], [7], [18], [21], 68 cases were selected based on the presence of focal genomic aberrations. Among the samples, 48 harbored focal genomic alterations either at 6p22, at 1q21–24, or both (Table S1). Alterations at 6p22 and 1q21–24 co-occurred in 18 samples. Alterations of the 6p22 and 1q21–24 region alone occurred in 11 and 19 samples, respectively, for a total of 29 samples with 6p22 alterations and 37 samples with 1q21–24 alteration. The 20 remaining samples lacking aberrations at 6p or 1q were selected based on the presence of other commonly recurring genomic alterations. Gene expression data was available for 212 of the original 261 samples, and for 58 out of the 68 samples selected for zoom-in analyses [6].

### Zoom-In array

A custom design 180 k Agilent G3 Sureprint (Agilent Technologies, Santa Clara, CA, USA) array was used, which covers the genome and contains increased probe densities at selected regions of the genome (Table S2). The average probe spacing was 17 bp and between 7000–12000 bp in selected target regions. Target regions were selected based on previous array CGH analyses using a 32 K BAC platform. Tumor sample and male reference DNA (Promega, Madison, WI, USA) were labeled and hybridized to arrays as described [22]. Tumor samples with a low DNA quantity were amplified using the GenomePlex WGA2 amplification kit (Sigma-Aldrich, St Louis, MO, USA) according to manufacturer's protocol with 20–40 ng of input DNA prior to labeling. The reference DNA for these samples was also subjected to whole genome amplification.

### Copy number analysis

Raw data was extracted from the scanned images using Agilent Feature Extraction 10.7.3.1 (Agilent Technologies, Santa Clara, CA, USA). The data was filtered from control probes and probes that did not pass Agilent's default "well above background" condition. Remaining probes were corrected for background signal and log 2 ratios (log 2 (Signal sample/Signal reference)) were calculated from the adjusted signal intensities for each array. The log 2 ratios were normalized and centered using popLowess [23]. The log 2 values of replicate probes were merged to their median value. Segmentation was performed on normalized log 2 ratios for each sample using Circular Binary Segmentation (CBS) [24] (Settings: 10 000 permutations, significance level for accepting change-points, α, set to 0.01, and a minimum of 5 consecutive probes for calling a segment). Gains and losses were called at regions where the segmentation value exceeded a sample adaptive threshold (SAT) [23]. The SAT ranged from 0.15 to 0.59, with a median value of 0.20. Copy number gain frequencies were calculated using segmented data at an individual probe level by dividing the number of times the probe was observed above the SAT with the number of samples investigated. Average copy number gain amplitudes (log 2) were calculated by measuring the summed segmentation line amplitude of each probe above SAT divided by the number of times the probe was observed above the SAT. RefSeq gene locations were downloaded from the UCSC genome browser (GRCh37/HG19 Assembly). MicroRNA (miRNA) data was obtained from miRBase (http://www.mirbase.org, Release 18). Copy number variant (CNV) data generated by Conrad *et al*. [25] was used to account for naturally occurring variations. Gene specific copy number was measured as the mean segmentation value spanning each RefSeq gene position. The correlation between gene specific copy number and gene expression levels was determined using Spearman correlation in the 58 samples with matched gene expression, and p-values were FDR corrected to account for multiple testing [26]. The gene expression levels in samples with amplifications were compared to the remainder of the 212 samples where expression data was available using the Mann-Whitney Test, in order to determine whether there was a significant difference in expression levels. Raw and processed data, together with array design and sample annotations, are deposited in the Gene Expression Omnibus (GSE40938).

### Breakpoint and sequence element analyses

Breakpoints were called at positions where the segmentation shifts exceed the SAT or occurred above the SAT. Breakpoints were manually curated in selected regions to account for outlier probes. In order to test for an uneven distribution of chromosomal breaks within the 1q and 6p target regions, the observed breakpoint distribution was compared to that of 10000 random permutations in 50 kb windows. Significance levels were determined by rank statistics. Data on repetitive genomic features (LINE, SINE, and LTR) was downloaded from the UCSC genome browser RepeatMasker track [27]. Locations of segmental duplications were obtained from the UCSC genome browser (Duplications of >1000 Bases of Non-RepeatMasked Sequence). G4 quadruplex locations were obtained using the Quadparser algorithm, which identifies d(G_3_N_1–7_G_3_N_1–7_G_3_N_1–7_G_3_) sequence motifs postulated to fold into a quadruplex structure [28]. LINE, SINE, LTR, and G4 sequence element content was measured in 50 kb non-overlapping windows across the genome. In order to assess the association between element content and breakpoint occurrence, the breakpoint frequency in windows that harbored an above median element content was compared to that of windows with a below median element content. Only regions with array coverage were included, and windows with CNVs were excluded. Fisher's exact test was used to assess the significance of repetitive sequence enrichment in the 1q and 6p amplicon peak regions.

## Results

### The 6p22 region

**Figure 1:**
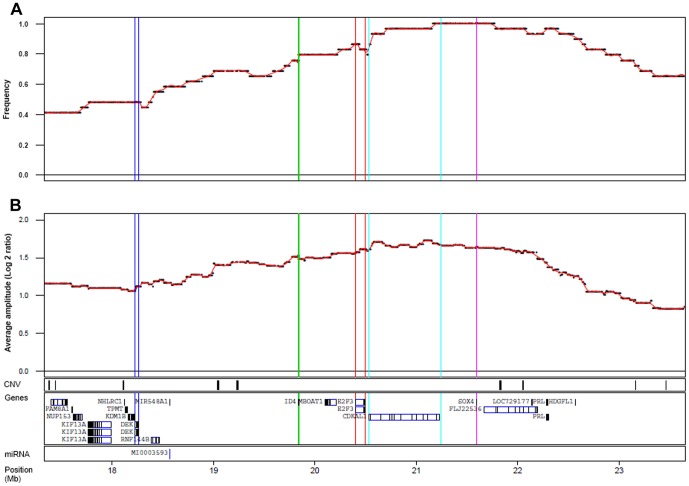
Summary of copy number gains at 6p22.3. A) Amplification frequency plot and B) average log 2 ratios for probes when amplified. Tracks for location of CNVs, genes, and microRNAs are given. Genomic positions in Mb (HG19).

**Figure 2:**
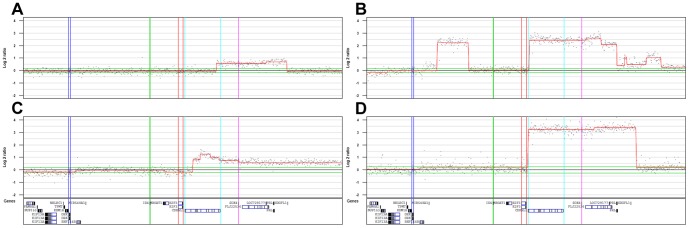
Focal 6p22.3 amplifications not including E2F3. Amplification breakpoints occur within the coding region of *CDKAL1* in A), B), and C), and within the *CDKAL1* promoter region in D).

Of the 261 cases analyzed by 32 K BAC array-CGH 29 cases showed focal copy number alterations occurring within the 6p22.3 region (Chr6:14.9–24.8 Mb). The frequency plot (Figure 1A) places *E2F3* at the slope of the amplification frequency peak, with the most frequently amplified gene being *SOX4*. When amplified, however, both genes show similar amplification amplitudes (Figure 1B). Although the focal genomic amplifications usually included all three genes (*E2F3, CDKAL1*, and *SOX4*), we detected four cases (14%) in which *E2F3* was not included in the amplified segments (Figure 2). These four cases showed amplification breakpoints between *E2F3* and *SOX4*: within the *CDKAL1* coding region in three cases and in the *CDKAL1* promoter region in one. Hence, the only intact amplified gene in these four cases was *SOX4*. No cases with *E2F3* amplification without concomitant *SOX4* amplification were found. This strongly argues for *SOX4* as an auxiliary target to *E2F3* in 6p22.

**Figure 3:**
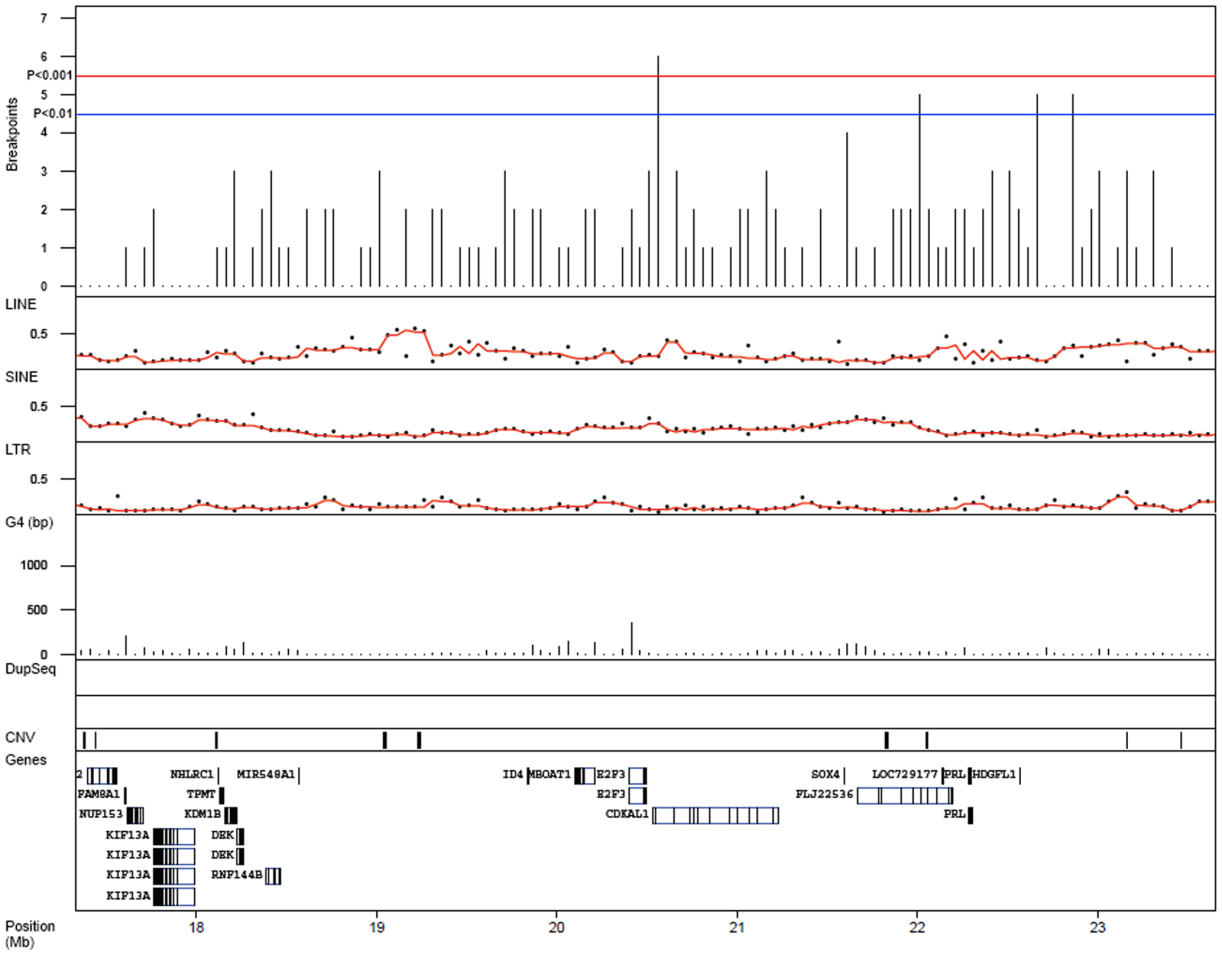
Chromosome 6p breakpoints. Breakpoint occurrence within 50 kb non-overlapping windows across the 6p target region. Significance thresholds, red line, p<10^−3^; blue line, p<10^−2^, determined by permutations (10000 fold) of breakpoints in the 6p22.3 region (Chr6:14.9–24.8 Mb). Tracks for LINE, SINE, LTR, and G4 element frequencies within 50 kb windows are given, as well as tracks for intraregional sequence duplications, CNVs, and genes. LINE, SINE, and LTR are displayed as percentage of window, while G4 is displayed as the number of base pairs of G4 sequence per window. No intraregional sequence duplications were located within the 6p22.3 peak region. Genomic positions in Mb (HG19).

A total of 213 segmentation shifts indicating chromosomal breaks were identified within the 6p22.3 region (Figure 3). The breaks were binned in 50 kb non-overlapping windows and tested for an uneven distribution within the region. Enrichment of breaks was observed between *E2F3* and *CDKAL1* (p<1×10^−3^) and to a lower extent at the proximal side of *SOX4* (p<1×10^−2^). To assess whether sequence elements were associated with breakpoint occurrence, the content of LINE, SINE, LTR, and G4 sequences was measured in 50 kb windows across the genome. The median genome-wide sequence element content per 50 kb window was 19.2% LINE, 10.8% SINE, 7.5% LTR, and 28 bp of G4 motif sequence. Genome-wide, breakpoints occurred preferentially in segments with an above median number of SINE and G4 elements, 1.8 and 1.4 fold higher frequency of breakpoints, respectively (p<3×10^−16^, Mann-Whitney test), and less frequently in segments enriched for LINE and LTR elements (0.8 and 0.8 fold, p<3×10^−16^). The 6p22.3 amplicon region showed a significantly higher frequency of SINE sequences but a significantly lower frequency of G4 sequences, compared to the genome as a whole (Table 1). No apparent association between breakpoints in the *E2F3-SOX4* region and the presence of the investigated sequence elements was observed (Figure 3).

**Table 1:** Summary of sequence element frequencies^1^.

Element	6p region^2^	6p vs WG^3^	1q region^2^	1q vs WG^3^	6p vs 1q^4^
LINE	55/125 (44.0%)	0.21	278/574 (48.4%)	0.47	0.38
SINE	77/125 (61.6%)	1.2 ×10^−2^	334/574 (58.1%)	9.4×10^−5^	0.55
LTR	62/125 (49.6%)	1	229/574 (38.1%)	1.6×10^−6^	5.7×10^−2^
G4	44/125 (35.2%)	1.2×10^−3^	356/574 (61.9%)	1.0×10^−8^	5.2×10^−8^

1 Values compared with whole genome median values ( = 50%).

2 Number of windows with above median number of repetitive sequence elements (6p region: Chr6:17.4–23.6 Mb, 1q region: Chr1:143.6–172.3 Mb, HG19).

3 p-values obtained by Fisher's exact test when comparing with the whole genome.

4 p-values obtained by Fisher's exact test when comparing frequencies in the 6p and the 1q region.

**Figure 4:**
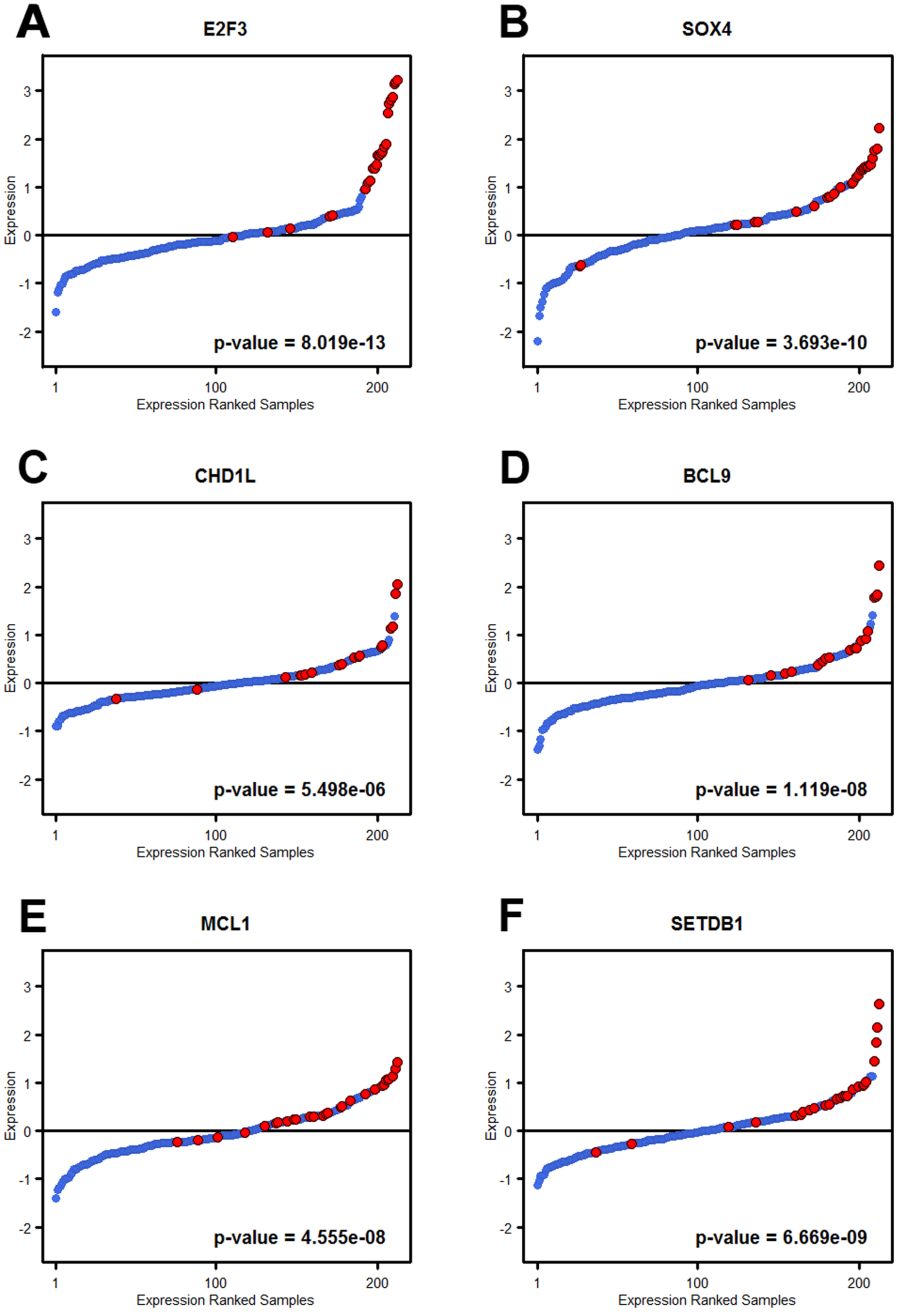
Association between gene amplification and expression. The 212 samples with both gene expression and genomic data were rank ordered based on A) *E2F3*, B), *SOX4* C) *CHD1L*, D) *BCL9*, E) *MCL1*, and F) *SETDB1* mRNA expression. Cases with focal genomic amplification of the respective gene are indicated with red. For each gene the difference in gene expression between amplified and non-amplified cases were tested by a Mann-Whitney test. The obtained p-values are indicated in each sub graph.

Correlations between DNA amplification and mRNA levels were found to be high for all genes within the amplified region, except for *ID4*. *MBOAT1* expression followed gene copy levels closely (ρ = 0.66, p<5×10^−6^) but was not always included in the amplified regions. *E2F3* showed strong correlation (ρ = 0.82, p<3×10^−16^) and the highest mRNA fold-changes. *SOX4*, the most proximal gene showed a highly significant association between gene copy numbers and gene expression (ρ = 0.59, p<8×10^−5^), as did *CDKAL1* (ρ = 0.78, p<3×10^−16^). *SOX4* was overexpressed in cases where *E2F3* was not a part of the amplicon. Hence, both increased *E2F3* and *SOX4* gene copy numbers are strongly associated with increased mRNA expression (Figure 4A and 4B).

### The 1q21–24 region

**Figure 5:**
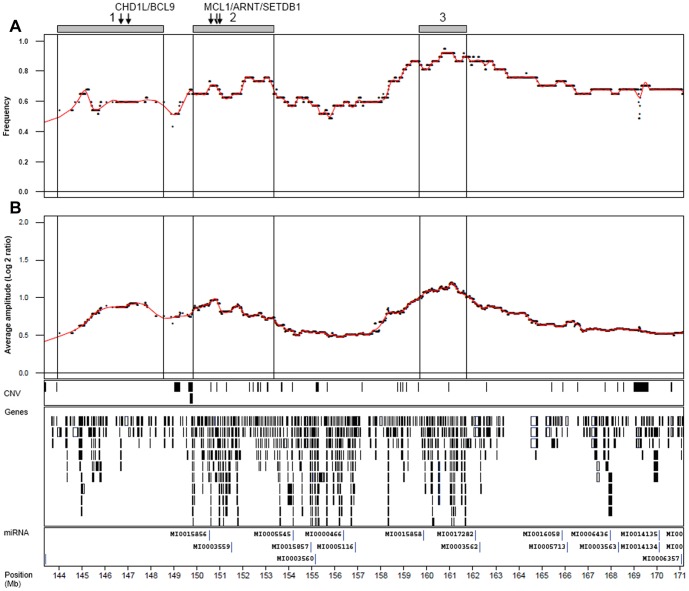
Summary of copy number gains at 1q21–24. A) Amplification frequency plot and B) average log 2 ratios for probes when amplified. Tracks for location of CNVs, genes, and microRNAs are given. Gray boxes indicate the extension of the three 1q amplicons. Arrows indicate the positions of CHD1L, BCL9, MCL1, ARNT, and SETDB1. Genomic positions in Mb (HG19).

**Figure 6:**
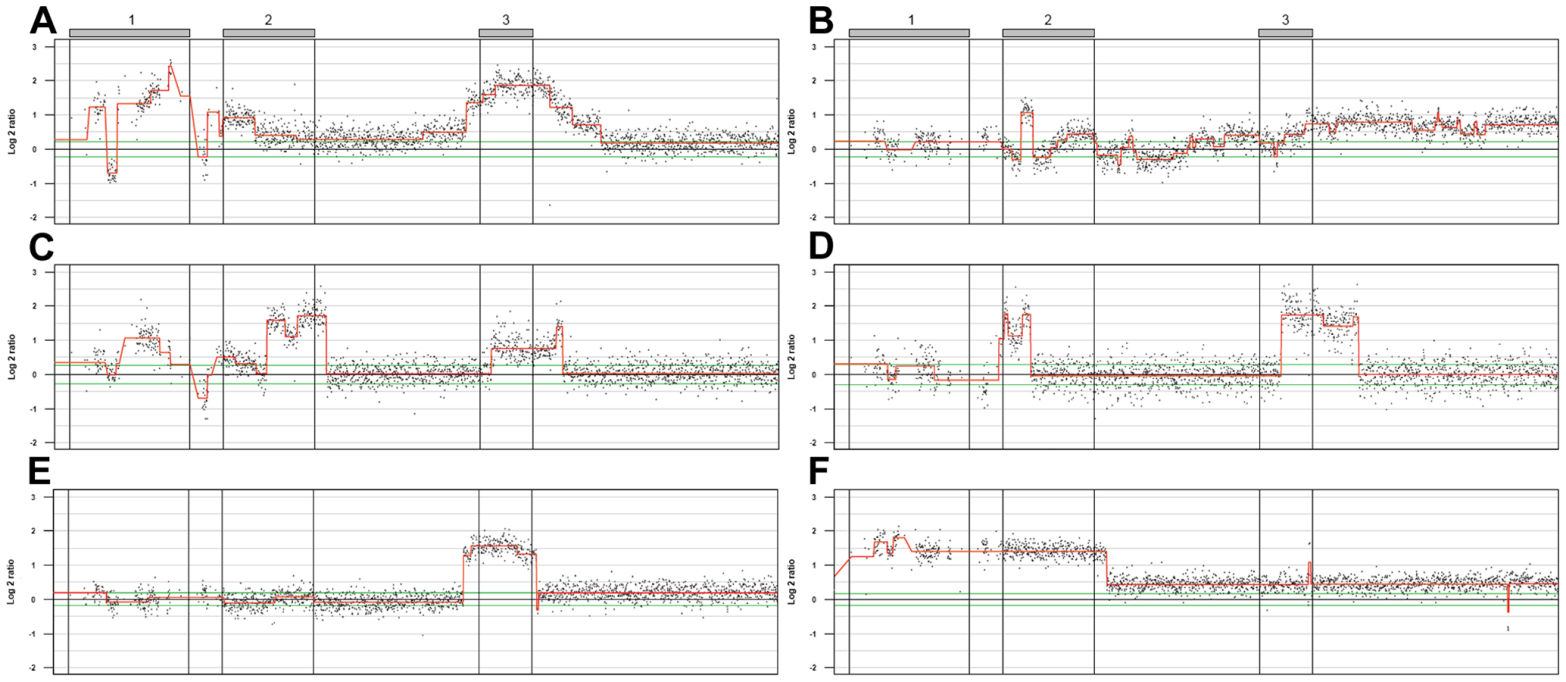
Examples of focal copy number gains within the 1q21–24 region. A) Each of the three amplicons amplified to a different extent, with a CNV loss occurring between amplicon 1 and amplicon 2. B) Amplicon 2 MCL1 region amplified. C) Similar event as in A but with varying copy number levels in amplicon 2. D) Amplicon region 2 and 3 amplified independently. E) Amplicon 3 amplified alone. F) Amplicon region 1 and 2 amplified as a single unit.

Thirty-seven of the 261 cases analyzed by 32 K BAC array-CGH harbored 1q copy number aberrations occurring within a 29 Mb genomic segment (Chr1:143.6–172.3 Mb). Although the high resolution zoom-in array further highlighted the heterogeneity of 1q alterations, three regions emerged as candidates for amplification: amplicon 1 at chr1:143.9–148.5 Mb, amplicon 2 at chr1:149.8–152.9 Mb, and a distal amplicon (amplicon 3) at chr1:159.7–161.7 Mb (Figure 5). These regions appear as concomitant amplifications in most cases: in 17 cases (46%) all three regions were amplified, in 6 cases (16%) amplicon 2 and 3, and in 2 cases (5%) amplicon 1 and 2. In no instance were amplicons 1 and 3 co-amplified without amplification of amplicon 2 (Figure 6). Only amplicon 3 was found amplified as a single unit, seen in 12 cases (34%).

Amplicon 1, observed in 19 of the 37 cases (51%) with 1q gains, always included the genes *BCL9* and *CHD1L*. A strong correlation between *BCL9* and *CHD1L* mRNA expression and gene copy numbers was also observed, (ρ = 0.63, p<2×10^−5^, and ρ = 0.53, p<2×10^−4^ respectively). Cases with amplified *BCL9* and *CHD1L* were highly enriched among the high expressing cases (Figure 4C and 4D). Amplicon 2 showed two possible sub-peaks that occasionally appeared as separate amplifications (Figures 6A, 6B, 6C and 6D). The anti-apoptotic gene *MCL1* was amplified in 25 out of the 37 (68%) cases, including one case with *MCL1* only. Two additional genes included in the peak region were: *ARNT*, also known as *HIF1B*, and *SETDB1*. *ARNT*/*HIF1B* was amplified in 24 (65%) of the cases, while *SETDB1* was amplified in 23 (62%) cases. All three genes showed a significant correlation between gene copy numbers and gene expression; *MCL1* (ρ = 0.73, p<3×10^−16^), *ARNT*/*HIF1B* (ρ = 0.54, p<3×10^−4^), and *SETDB1* (ρ = 0.64, p<6× 10^−6^). Cases with *MCL1*, and *SETDB1* amplifications where highly enriched among the high expressing cases (Figure 4E and 4F), as was *ARNT*/*HIF1B* (not shown). The third amplicon region harbored copy number aberrations in 35 out of 37 cases (95%). The amplicon region spans approximately 68 genes but the amplification frequency peaks around 25 genes located at chr1:160.84–161.35 Mb (Table S3). Eleven of these genes showed strong association (ρ≥0.55, p<3×10^−4^) between gene copy number and gene expression (Table 2), including the tight junction adhesion related *F11R*, the death effector domain containing *DEDD*, and the transcription factor *USF1*, as well as four genes associated with mitochondrial functions: *PPOX*, *NDUFS2*, *TOMM40L*, and *SDHC*.

**Table 2:** Correlation between gene copy numbers and gene expression.

Gene	Correlation^1^(ρ)	p-value
*F11R*	0.63	<2×10^−5^
*USF1*	0.59	<7×10^−5^
*NIT1*	0.58	<8×10^−5^
*DEDD*	0.77	<3×10^−16^
*UFC1*	0.61	<3×10^−5^
*USP21*	0.67	<3×10^−6^
*PPOX*	0.71	<3×10^−16^
*B4GALT3*	0.80	<2×10^−11^
*NDUFS2*	0.64	<9×10^−6^
*TOMM40L*	0.55	<3×10^−4^
*SDHC*	0.75	< 3×10^−16^

1 Spearman rank correlation.

**Figure 7:**
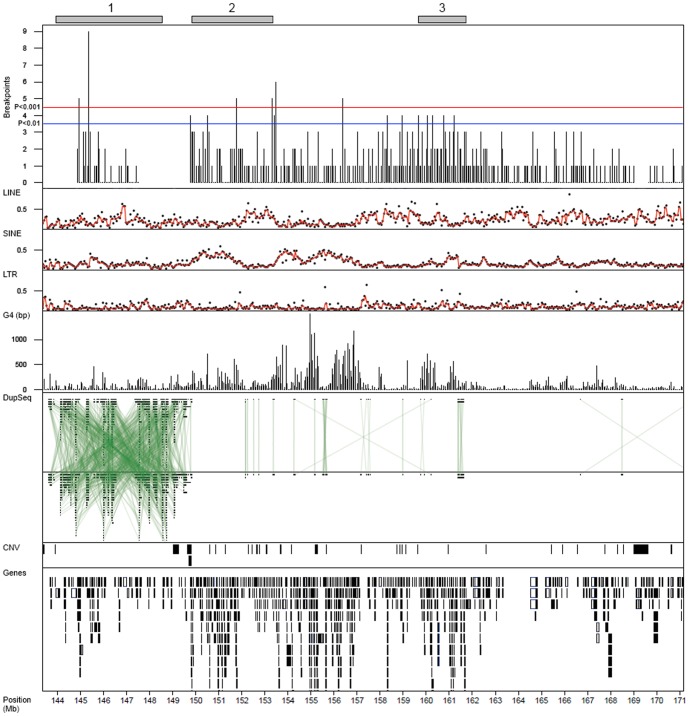
Chromosome 1q breakpoints. A) Breakpoint occurrence within 50 kb non-overlapping windows across the 1q target region. Significance thresholds, red line, p<10^−3^; blue line, p<10^−2^, determined by permutation (10000 fold) of breakpoints in the 1q region (Chr1:140.0–184.0 Mb). Tracks for LINE, SINE, LTR, and G4 element frequencies within 50 kb windows are given. LINE, SINE, and LTR are displayed as percentage of window, while G4 is displayed as the number of base pairs of G4 sequence per window. Intraregional sequence duplications are connected with green lines in the DupSeq track. Locations of CNVs and genes are given in individual tracks. Genomic positions in Mb (HG19).

A total of 599 segmentation shifts indicating chromosomal breaks were detected within the 1q amplification region (Figure 7). One region, located within amplicon 1, showed a strong enrichment for breakpoints (p<10^−4^). No clear association between the clustering of breakpoints and specific sequence elements could be established. However, compared to the whole genome, the 1q region shows higher frequencies of G4 and SINE elements, and lower frequencies of LTR sequences. Furthermore, the 1q region differed significantly from 6p amplification regions with respect to G4 element content (Table 1). A notable feature of the 1q region is the high frequency of intraregional sequence duplications (Figure 7), particularly within the amplicon 1 segment. Similar occurrences of intraregional sequence duplications were not observed in the 6p region (Figure 3).

## Discussion

The most frequent genomic copy number gains in UC occur on 6p and 1q. The 6p amplification, mostly seen in high grade tumors, has been extensively studied and *E2F3* is believed to be the main target. There are however cases with 6p amplifications that do not cover *E2F3* [7]. Aberrations of 1q occur both in low and high grade tumors. However, whereas whole chromosome arm gains are seen in low grade tumors, high grade tumors frequently show complex focal amplifications [7], [19]. In addition, no bona fide target genes have so far been assigned to the 1q region in UC. To resolve some of these issues we selected 29 cases with 6p22 and 37 cases with 1q21–24 focal amplifications from a series of 261 cases analyzed by 32 K BAC array-CGH for high resolution zoom-in array CGH analyses. The applied zoom-in platform has an approximately ten-fold increase in resolution with a design that makes it possible to identify intragenic breakpoints.

The abundance and the high sequence similarity among repetitive elements make them potential driving factors for genomic instability [29]. Mechanisms suggested to be in operation include un-equal crossing-over and non-allelic homologous recombination repair events [30]–[33]. Both the 6p and the 1q regions contained higher frequencies of SINE elements that may contribute to the nature of the amplifications. Alternative forms of secondary DNA structures have also been linked to genomic instability [34]–[38] such as G4 quadruplexes, formed by guanine-rich sequences that adopt four-stranded secondary DNA structures [39]. Regions rich in G4 sequence motifs have been shown to be enriched for DNA breaks in cancer [38], something we also observe in the present study. Furthermore, hypomethylation, a common feature of cancer genomes, potentially aids the formation of G4 quadruplex structures [38]. In contrast to 6p, the 1q region showed a high frequency of G4 quadruplex sequence motifs, particularly in the amplicon regions 2 and 3. Amplicon 1, on the other hand, showed a large number of intraregional sequence duplications, a feature that is absent in the 6p region. Hence, our data suggest that the observed heterogeneity of 1q amplifications may be a consequence of an underlying regional instability caused by an accumulation of specific sequence motifs. Regions with similarly high density of regional sequence duplications are also seen in other peri-centromeric regions e.g., in chromosomes 7, 9, and 16.

Several investigations have indicated *E2F3* as the major target gene for 6p22 amplifications [40]–[44]. E2F3 has a central role in cell cycle regulation [45] and the frequent *E2F3* amplifications are consistent with the frequent *RB1* alterations seen in UC, both affecting the same key transition in cell cycle regulation [46]. Hurst et al. [40] have pointed to an intimate link between E2F3 and RB1 in UC and we have recently identified an *E2F3*/*RB1* genomic circuit operating in a subset of UCs [7]. In light of this, it is intriguing that *E2F3* is not the most frequently amplified gene at 6p22. The finding of 6p22 amplifications not spanning the *E2F3* gene, with genomic breaks within the *CDKAL1* gene, strongly suggests *SOX4* as possible auxiliary target gene within 6p22. Intriguingly, both depletion and overexpression of *SOX4* may have unfavorable effects on cell survival [47], [48]. Recent investigations have reported SOX4 as a part of the pro-apoptotic TP53 pathway in which *SOX4* expression is induced during DNA damage and stabilizes TP53 by blocking MDM2-mediated ubiquitination and degradation [49]. This function could explain why *SOX4* overexpression has been linked to apoptosis and been associated with better patient survival [47], [50]. In contrast to these findings, *SOX4* has also been reported to have positive effect on cellular survival [48], [51]. *SOX4* expression has been linked to increased proliferation through modulation of β-catenin/TCF activity in *TP53* mutated cell lines [52]. In addition, *SOX4* expression activates *EGFR* expression and influences the NOTCH pathway [52], [53]. Taken together these findings indicate SOX4 as a multifunctional protein that may have a context dependent cellular function. All four cases with *SOX4* but not *E2F3* amplification harbored *TP53* mutations. This leaves the question open whether *SOX4* could have oncogenic properties when amplified in *TP53* mutated cases of UC. Recent investigations have shown that SOX4 is regulated through rapid protein degradation [54]. This indicates that SOX4 function may, in analogy with TP53, be required or triggered at specific cellular conditions or transitions. As a consequence, *SOX4* gene copy number alterations resulting in increased mRNA levels does not necessarily have to result in increased steady state SOX4 protein levels. Accordingly, our attempts to establish a link between *SOX4* gene copy numbers and increased protein levels by IHC did not show any convincing results. This does however not exclude an oncogenic potential of the SOX4 protein.

Even though many studies identify 1q amplifications as a frequent event in UC, few studies report on specific target genes. This is probably due to the fact that the 1q target region is large and gene dense, and as a consequence, may harbor several target genes. Furthermore, 1q amplifications are heterogeneous and occur in a large genomic region, spanning more than 29 Mb. At least three regions could be identified based on the copy number frequency profiles in the current study. The most proximal region was amplified in close to 60% of the cases with 1q alterations. This region contains at least two genes with potential tumor promoting characteristics: *BCL9* and *CHD1L*. BCL9 acts as a nuclear component of the Wnt pathway in association with LEF/TCF family members [55]. *BCL9* overexpression has been linked to increased tumor cell proliferation, survival, migration, and invasion by enhancing β-catenin-mediated transcriptional activity [56], [57]. Furthermore *BCL9* knock-down tumors show a less aggressive phenotype and result in increased host survival in mouse xenograft models of multiple myeloma and colon carcinoma [56]. Overexpression of *CHD1L*, also known as *ALC1 (amplified in liver cancer 1)*, has been found to inhibit apoptosis, promote G1/S transition, and promote tissue invasion and metastasis [58]–[60]. Furthermore, *CHD1L*-transgenic mice develop spontaneous tumors in various organs, including liver, neck, and colon [61]. Hence, increased expression of both *BCL9* and *CHD1L* may have tumor promoting effects. The analysis highlighted three genes within the central amplified region on 1q: *MCL1*, *ARNT/HIF1B*, and *SETDB1*. *MCL1* is a member of the *BCL2* anti-apoptotic gene family and a part of a commonly amplified region containing at least six additional genes that are altered in several cancer types [62]. siRNA knockdown of *MCL1* results in increased apoptosis, clearly indicating *MCL1* as a target for amplification [62]. HIF1B forms a hetero-dimer with HIF1A and EPAS1/HIF2A that functions as a transcriptional regulator of the adaptive response to hypoxia [63], [64]. Adaptation to hypoxic conditions may be a prerequisite for tumor progression and metastasis [65]. A recent large-scale study identified a region spanning from *MCL1* to *SETDB1*, as a key amplified region in malignant melanoma, and suggested *SETDB1* as the target gene [66]. This was motivated by the finding that overexpression of *SETDB1* in an animal model resulted in accelerated melanoma onset and formation [67]. The *SETDB1* gene was, however, not always included in the 1q amplifications in the present cohort of UCs. The best established oncogene of the three genes in the central amplicon is *MCL1* [62]. As SOX4, MCL1 protein is rapidly degraded by the proteasome which makes an association between gene copy numbers and protein expression hard to establish [68]. However, cells with *MCL1* amplification show a more pronounced response to shRNA knock-down of *MCL1* than cells wild-type for the gene [62]. In conclusion our analysis of the 1q and 6p regions highlights intrinsic features of the genome such as repetitive element and G4-sequence content as putative enablers of chromosomal instability. The stark contrast between the 1q and 6p amplification patterns suggests that different mechanisms and selection pressures may dictate the appearance of the respective genomic alterations. Further studies are needed to resolve the question of whether the heterogeneous appearance of the 1q region is the result of complex rearrangements in an unstable region or the result of clonal heterogeneity at the population level.

## Supporting Information

Table S1 **Stage, Grade, and aberrations.** Information on stage, grade and genomic aberrations for the samples included in the study.
(XLSX)

Table S2 **Regions of increased array coverage, based on frequently occurring alterations in UC.**
(XLSX)

Table S3 **Correlation between gene copy number and gene expression for genes within amplicon region 3 (Chr1:159.7–161.730 Mbp, HG19).**
(XLSX)
